# Report from the First Latin American Urological Oncology Symposium (SLAURO) 19–21 June 2014, Viña del Mar, Chile

**DOI:** 10.3332/ecancer.2014.ed47

**Published:** 2014-11-27

**Authors:** Christian Caglevic, Ivàn Pinto, Jaime Altamirano, Roberto Vilches, Eu Marìa Eliana San Martìn, Jorge Gallardo

**Affiliations:** 1 Medical Oncologist, Fundación Arturo López Pérez [Arturo López Pérez Foundation], Santiago de Chile 7500921, Chile; 2 Urologist, Past Chief of Urology, Fundación Arturo López Pérez, Santiago de Chile 7500921, Chile; 3 Urologist, Resident in Oncological Urology, Fundación Arturo López Pérez, Santiago de Chile 7500921, Chile; 4 SLAURO Executive Director 2014, Fundación Cáncer Chile [Chilean Cancer Foundation], 7501088, Chile; 5 Medical Oncologist Clínica Alemán de Santiago de Chile [Santiago de Chile German Clinic], Fundación Cáncer Chile President, 7501088, Chile

## Abstract

Cancer is one of the most important diseases in Chile, with alarming incidence and mortality rates that are among the highest in Latin America. Economic growth in South America has led to demographic change, with an aging population typical of developed countries, but also a growing population with cancer. The incidence and mortality of urological cancers in Chile is significant, and has led to the formulation of health laws and policies promoting the early treatment of urological cancers. It is also well known that there are regions of Chile with extremely high incidence and mortality of bladder cancer caused by arsenic exposure. SLAURO (*Simposio Latinoamericano de Urología Oncológica* [Latin American Oncological Urology Symposium]) is a new Latin American forum for discussing and promoting knowledge of urological cancers across the region.

## Urological cancers in Chile – Background

Chile is transitioning from an underdeveloped economy to a developed one [1]. This transition involves important variations in population curves, with a significant rise in the number of people over the age of 60 [2]. Dietary and self-care habits are changing as the population ages, leading to increasing obesity and chronic diseases. Unfortunately, tobacco use also remains extremely high in Chile, despite the efforts of health policymakers. Chilean tobacco users start smoking as early as their teenage years, and smoking is unquestionably associated with the onset of various types of cancers [3].

Chile lacks good statistical cancer records, but there are published data showing that the country has some of the highest cancer incidence and mortality rates, not only in Latin America, but also in the world. The currently accepted incidence figures are 240 cancers per 100,000 in residents, with 120 deaths per 100,000 residents. There are predictions that cancer will surpass cardiovascular disease as a cause of death in Chile around the year 2030. Incidence and mortality rates in other South American countries may be similar to those in Chile, but we can’t be certain given the lack of statistical records about cancer [4–5].

The magnitude of cancer incidence in Chile has required the development of public policies that promote earlier screening and effective treatments for various cancers. Urology-related cancers are definitely among the targets of these screening and treatment policies.

Chile essentially has two health systems: a governmental system that covers the majority of Chileans: the FONASA (*Fondo Nacional de Salud* [National Health Fund]) System and a ‘private’ system, ISAPRES (*Instituciones de Salud Previsional* [Chilean Health Plan Providers]), which covers mainly those with greater financial means [6]. Regardless of which system Chileans belong to, the Explicit Health Guarantees Law [GES in Spanish], guarantees them timely care for various diseases [7], including three urological cancers: prostate, testicular, and bladder cancer.

The Chilean public health system cared for over 2,000 patients with testicular cancer between 1988 and 2007 (including primary extragonadal carcinomas). The average age of those patients was 29 years for patients with non-seminomas (36% of testicular cancers in Chile) and 35 years for patients with seminoma (64% of testicular cancers in Chile). Timely diagnosis and treatment of testicular cancer can save an extremely high percentage of patients with the disease. Late treatment, however, is associated with high mortality and an increase in potential lost working years [8].

Prostate cancer is another disease that affects only men. We know that 70% of autopsies of men aged 80 years and older detect prostate cancer. The average time from diagnosis to the onset of metastasis, however, is five years, and death from prostate cancer usually occurs 10 years after initial diagnosis. According to official statistics, approximately 1,200 Chileans die of prostate cancer annually. These figures also show an incidence of 55–57 cases per 100,000 male residents annually and a prevalence of 9.2 cases per 1,000 men aged 40 to 59 [9]. It is highly likely that, in light of early prostate cancer screening campaigns, these figures are of great significance. Chile’s demographic transition and lifestyle changes, however, must be having an important effect on prostate cancer incidence and prevalence rates.

Bladder cancer in Chile is a special case [10]. The south-central region of the country has bladder cancer incidence rates similar to those of developed countries. Official figures for the years 1998–2002, however, show that northern Chile, and particularly the Antofagasta region, had an incidence of nearly 25 cases of bladder cancer per 100,000 residents (more than five times greater than the incidence in southern Chile). We cannot rule out *a priori* that there has been an increase since 2002. Bladder cancer affects two to three times as many men as women in Chile, and researchers have correlated the incidence of bladder cancer in Northern Chile with exposure to arsenic, mainly in drinking and irrigation water [11].

The aforementioned figures and the lack of international discussion forums dealing with urological cancers in Chile and some Latin American regions highlighted the need for a forum that would gather specialists who could meet and discuss these diseases.

The Latin American Oncological Urology Symposium (SLAURO) is the first transnational and multidisciplinary forum in Latin America for the study, treatment, and follow-up of oncological urological disease [12]. The most recent SLAURO symposium was organized by the Chile Cancer Foundation and led by Dr. Francisco Orlandi.

SLAURO took place from June 19–21, 2014, in the coastal city of Viña del Mar, Chile’s main beach resort. The symposium drew 247 professionals, including urologists, radiation oncologists, radiologists, medical oncologists, and nurses who confront urological cancers daily in their clinical practices. Organizers divided the symposium into various thematic sessions, offering the invited guests and other attendees sessions covering renal cancer, testicular cancer, bladder cancer, and prostate cancer. Each session featured national and international experts offering personal presentations, roundtable discussions, or symposia organized by SLAURO sponsors.

The renal cancer session featured Dr. Mauricio Burotto, a Chilean researcher currently at the National Cancer Institute (Bethesda, Maryland, U.S.). Dr. Burotto explained his work in the field of renal cancer, new tumor growth constant models, and their application in monitoring patients with metastatic renal cancer. These models have contributed to progress in the standardization and precision of studies assessing new systemic therapies. Unlike earlier models, they will allow us to anticipate response to treatment.

The renal cancer session also featured international guest Dr. Gilberto Schwartsmann of the Federal University Academic Hospital in Porto Alêgre, Brazil. Dr. Schwartsmann presented an updated overview of systemic treatment of metastatic renal cancer, and explained the current treatment standard as well as new research challenges in this field. Dr. Schwartsmann also highlighted studies by Dr. Motzer, which have presented PAZOPANIB as therapeutically equivalent to SUNITINIB, but with a better tolerance profile and quality of life. Dr. Schwartsmann also highlighted Nivolumab as a second-line therapy for renal cancer (ASCO [American Society of Clinical Oncology] 2014).

The testicular cancer session featured Dr. Silvia Zunino, a radiation oncologist at the Instituto de Radioterapia Fundación Marie Curie [Marie Curie Foundation Radiation Oncology Institute] (Cordova, Argentina). Dr. Zunino highlighted the impact of new technologies in radiation oncology planning, which have led to lower dosages and toxicity, improving safety profiles in patients with stage 1 seminomas.

The presentation and introduction of new pharmacological alternatives for patients with castration-refractory prostate cancer was unquestionably one of the most notable and innovative features of the symposium. We were fortunate that guest speaker Dr. Roland de Wit came from the Erasmus Cancer Institute in Rotterdam, Holland. Dr. de Wit explained avenues of research on optimal pharmacological sequencing of the various treatment lines in castration-resistant prostate cancer. As an example, Dr. de Wit explained the importance of early Docetaxel use in patients with a high tumor load at diagnosis. He then covered how to ensure the compatibility of Docetaxel and other pharmacological therapies.

One of the Chilean speakers, Dr. Iván Pinto, Chief of Uro-Oncology at the Fundación Arturo López Pérez, presented the safety profile and role of Abiraterone use in patients with non-metastatic castration-resistant prostate cancer. Dr. Pinto also highlighted a series of patients who had rescue lymphadenectomy following curative-intent treatment for isolated recurrent pelvic lymph nodes detected by choline PET/CT scan. Half of these patients are in complete disease remission.

We also wish to note a social highlight of the symposium, the presentation of an award honoring the career of Dr. Octavio Castillo, one of the leading urologists specializing in robotic and minimally invasive surgery in Latin America. This award recognized Dr. Castillo’s contributions to the field of urological oncology.

In conclusion, we hope that SLAURO will grow as a scientific and academic forum for Latin America and for the world of urology. We also hope that we will be able to welcome even more national and international attendees at the next symposium.
